# *Eupatorium fortunei* and Its Components Increase Antiviral Immune Responses against RNA Viruses

**DOI:** 10.3389/fphar.2017.00511

**Published:** 2017-08-03

**Authors:** Jang-Gi Choi, Heeeun Lee, Youn-Hwan Hwang, Jong-Soo Lee, Won-Kyung Cho, Jin Yeul Ma

**Affiliations:** ^1^Korean Medicine Application Center, Korea Institute of Oriental Medicine Daegu, South Korea; ^2^College of Veterinary Medicine, Chungnam National University Daejeon, South Korea

**Keywords:** *Eupatorium fortunei*, quercetin, antiviral effect, RNA viruses, herbal medicine

## Abstract

*Eupatorium fortunei* (EF) has long been used as herbal medicine in Korea, China, and Asian countries to treat a variety of diseases. Recent studies have reported that EF has anti-metastatic, anti-angiogenic, anti-bacterial, and anti-oxidant activities, as well as activities against malignant metastatic human cancers. The effect of EF and its components on viruses has not been reported. In the present study, the antiviral activity and mechanism of action of an aqueous extract of EF (WEF) and its components were evaluated *in vitro*. We found that pretreatment with WEF markedly reduced viral replication, as evaluated using a green fluorescent protein (GFP)-tagged virus (influenza A virus, Newcastle disease virus, and vesicular stomatitis virus) in murine RAW 264.7 macrophage cells. We demonstrated that WEF induces the production of type I IFN including pro-inflammatory cytokines. Additionally, we identified the active anti-viral components of WEF as quercetin, psoralen, and quercitrin. Thus, WEF and its active components are immunomodulators of the innate immune response in murine macrophages, a finding that is potentially useful to developing prophylactic or therapeutic treatments against a range of viruses.

## Introduction

Viral pathogens cause malignant diseases in humans and animals, resulting in considerable mortality, morbidity, and economic losses worldwide (Thompson et al., 2002; Lindahl and Grace, 2015). Vaccines and treatments for various diseases have been developed, but new mutant strains resistant to these vaccines emerge (Moscona, 2005). The rapid emergence of several new drug-resistant viruses necessitates the development of new treatment modalities (Moscona, 2005), including effective chemotherapeutic agents (Hurt et al., 2012; Choi et al., 2016; Piret and Boivin, 2016).

Herbal medicine, which is a traditional medicine used in Korea, China, and Asian countries, has been used for thousands of years as a combination of various compounds. The method of taking traditional medicine typically involves oral administration as water extracts. In addition, traditional medicine has relieved the symptoms of various diseases including cancer and infectious diseases, and has been used for thousands of years and is still being used. Several herbal medicines have been used to prevent and treat virus infections and immune enhance immunity (Cha et al., 2007). Currently, research is being conducted worldwide to search for antiviral materials using traditional medicinal plants. In addition, the emergence of tolerance to existing antiviral agents calls for the development of new candidates, and there is an increasing interest in the development of antiviral agents using traditional medicinal plants. Expensive antiviral agents are still a burden on developing countries, and effective and cheap antiviral agents are one of the world's top priorities for drug development. In addition, natural products derived from medicinal plants play a role as one of the most important raw materials of lead compounds in pharmaceuticals. Therefore, the search for antiviral active substances using traditional medicinal plants offers great potential for the development of potentially effective new antiviral agents (Lin et al., 2014; Talactac et al., 2015).

*Eupatorium fortunei* (EF) has long been used for the management of fever, edema, and chills (Kim et al., 2014). EF is reported to possess anti-oxidant, anti-diabetic, and anti-bacterial activities and to have anti-metastatic and anti-angiogenic effects on malignant human cancer cells (Kim et al., 2014). In this study, we investigated the induction of immunomodulatory signaling molecules by the water extract of EF (WEF). We also sought to identify the active components of WEF using UPLC-MS/MS. In addition, we evaluated the efficacy of WEF against influenza A virus, new castle disease virus, and vesicular stomatitis virus *in vitro*.

## Materials and methods

### Preparation of WEF

The WEF was prepared as described previously (Kim et al., 2014). Dried EF was purchased from Yeongcheon Oriental Herbal Market (Yeongcheon, South Korea) and stored in the KM-Application Center herbarium (registration number, #354) Korea Institute of Oriental Medicine(KIOM), after verification of identity by Prof. Ki Hwan Bae (Chungnam National University, Daejeon, Korea). The WEF was filtered through standard sieves (150 μm, Retsch, Haan, Germany), concentrated by lyophilization, and stored at −20°C until use (Cho et al., 2015).

### Cells and viruses

Madin-Darby Canine Kidney (MDCK, ATCC CCL-34, NBL-2), African green monkey kidney (Vero, ATCC CCL-81), and RAW264.7 (murine macrophage, ATCC TIB-71) cells were cultured in Dulbecco's Modified Eagle's Medium (DMEM; Lonza, Walkersville, MD) containing 10% fetal bovine serum (FBS; Cellgro, Manassas, VA, USA) and 1% antibiotics (100 U/ml penicillin and 100 μg/ml streptomycin) at 37°C with 5% CO_2_ as described previously (Cho et al., 2015). Influenza A/PuertoRico/8/34 H1N1, green fluorescent protein (GFP)-tagged Influenza A/PuertoRico/8/34 H1N1 (PR8-GFP), and NDV-GFP were propagated in the allantoic fluid of 10-day-old chicken embryos. VSV-GFP was propagated on confluent Vero cells (Cho et al., 2015).

### MTS assay

Cell viability was measured after 24 h of incubation in WEF (0–2,000 μg/mL) using the MTS assay (Promega, Madison, WI, USA), according to the supplier's instructions (Choi et al., 2016). Briefly, RAW 264.7 (1 × 10^5^ cells/well) cells seeded in 96-well tissue culture plates were incubated for 12 h at 37°C with 5% CO_2_. WEF was added, and the cells were incubated for 24 h. Ten microliter of MTS solution was added to each well, and the cells were incubated for additional 2 h. The absorbance at 490 nm was recorded using the Glomax Explorer System (Promega, Madison, WI, USA) (Choi et al., 2016).

### Viral replication inhibition assay

A viral replication inhibition assay was performed using the GFP viruses described previously (Cho et al., 2015; Choi et al., 2016). We tested the antiviral effect of WEF against viral pathogens previously used in studies as challenged viruses, such as VSV, NDV, and PR8-GFP (Talactac et al., 2015). RAW264.7 cells (8 × 10^5^ cells/well) were seeded in 12-well plates, and the cells were incubated for 12 h. Cells were incubated for 12 h in DMEM alone (untreated or virus-only group), DMEM with 1,000 U of recombinant mouse interferon (IFN-β, positive control), or DMEM with 100 μg/mL WEF. The cells then were infected with PR8-GFP (MOI 1), NDV-GFP (MOI 2), and VSV-GFP (MOI 1). GFP expression was measured at 12 and 24 h post-infection (hpi) at 200× magnification and was measured 24 hpi using the Glomax multi-detection system (Promega, Wisconsin, USA) as per the manufacturer's instructions (Cho et al., 2015; Talactac et al., 2015).

### Enzyme-linked immunosorbent assay (ELISA)

The levels of murine IL-6 (BD Bioscience, USA), TNF-α (BD Bioscience, USA), and IFN-β (PBL Interferon Source, USA) in culture supernatants were measured using ELISA antibody kits following the manufacturer's instructions (Wadsworth and Koop, 1999; Talactac et al., 2015).

### Quantitative real-time PCR

Total RNA was isolated from RAW 264.7 cell lysates using an RNeasy mini kit (Qiagen, Hilden, Germany) following the manufacturer's instructions. Total RNA (1,000 ng) was used to synthesize cDNA using the RevoScript RT PreMix (iNtRON Biotechnology, Sungnam, Korea). qRT-PCR was performed using the primers listed in Table 1 and AccuPower® 2× Greenstar qPCR Master Mix (Bioneer, Daejeon, Korea). Relative expression was calculated using the ΔΔCt method and β-actin (internal reference) to normalized mRNA expression levels (Choi et al., 2016).

**Table 1:** Primer sequences for quantitative real-time RT-PCR.

**Name**	**Orientation**	**Primer sequences (5′to 3′orientation)**
β-Actin	Forward	AGGTGTGCACCTTTTATTGGTCTCAA
	Reverse	TGTATGAAGGTTTGGTCTCCCT
HA	Forward	TTGCTAAAACCCGGAGACAC
	Reverse	CCTGACGTATTTGGGCACT-
M2	Forward	GAAAGGAGGGCCTTCTACGG
	Reverse	TCGTCAGCATCCACAGCAC
NP	Forward	GAATGGTGCTCTCTGCTTTTGA
	Reverse	TCCACTTTCCGTTTACTCTCCTG
PA	Forward	AAGTGCCATAGGCCAGGTTTC
	Reverse	CCTCATCTCCATTCCCCATTTC
PB2	Forward	GGTGCTTACGGGCAATCTTC
	Reverse	TGTTCGTCTCTCCCACTCACTATC
NS-1	Forward	GCGATGCCCCATTCCTTG
	Reverse	ATCCGCTCCACTATCTGCTTTC-

### Immunofluorescence staining

RAW 264.7 cells (1 × 10^5^) seeded onto four-well tissue culture slides were cultured at 37°C with 5% CO_2_ for 24 h. WEF, IFN-β, quercetin, and psoralen were then added to the cells, and the cells were incubated at 37°C with 5% CO_2_ for 12 h. The medium was then removed, and the cells were washed with cold PBS three times and infected with A/PuertoRico/8/34 (MOI 1) for 2 h. After viral infection, the virus and medium were removed, and the cells were washed with PBS three times. Complete medium was then added to cells, and the cells were incubated at 37°C with 5% CO_2_ for 24 h. After 24 h, cells were washed with cold PBS and fixed with 4% paraformaldehyde for 30 min at RT and permeabilized with 1% Triton X-100 in TBS for 15 min at RT. After blocking, cells were incubated with a rabbit polyclonal antibody detecting M2 (diluted 1:1,000 in TBS buffer; GeneTex, San Antonio, TX, USA) at 4°C overnight, washed with TBS three times, and incubated with a Alexa Fluor 568 goat anti-rabbit IgG antibody (diluted 1:1,000 in TBS buffer; Thermo Fisher Scientific, Waltham, MA, USA) for 1 h. Nuclei were visualized by staining with DAPI (1 μg/mL) for 10 min. Images were captured using a fluorescence microscope (Olympus, Tokyo, Japan).

### Western blot analysis

The RAW 264.7 cells (1 × 10^6^ cells/6-well) were incubated with WEF (1, 10, and 100 μg/mL), LPS (100 ng/mL), and IFN-β (1,000 units) at 37°C with 5% CO_2_. After 24 h, cells were harvested at the indicated times (4, 8, 12, and 24 h). Proteins in the RIPA buffer cell lysates (20 μg) were separated by 12% SDS-PAGE and transferred to a polyvinylidene difluoride (PVDF) membrane. The membrane was then blocked with 5% BSA for 1 h and incubated at room temperature for 1 h with primary antibodies (1:1,000 dilution) against anti-IRF3, anti-STAT1, anti-TBK1, anti-phospho-IRF3, anti-phospho-STAT1, and anti-phospho-TBK1 antibodies (Cell Signaling Technology, Boston, MA, USA) and β-actin (Cell Signaling Technology, Boston, MA, USA). After washing the blot in TBS three times, HRP-conjugated secondary antibodies (1:2,000 dilution) were incubated at RT for 1 h. Protein bands were visualized using a chemiluminescent reagent (Thermo Scientific, Waltham, MA, USA), and the relative intensities of protein bands were measured using Image J.

### Identification of phytochemical constituents

Fifteen authentic standards were used for the identification of phytochemicals in WEF. Psoralen (1), angelicin (2), rutin (3), quercitrin (4), isoquercitirin (5), syringin (9), caffeic acid (10), 1,3-dicaffeoylquinic acid (11), sweroside (15), and scopoletin (internal standard) were purchased from ChemFace (Wuhan, China). Quercetin (6), neochlorogenic acid (7), chlorogenic acid (8), *p*-coumaric acid (12), ferulic acid (13), and *o*-coumaric acid (14) were obtained from Sigma-Aldrich Co. (St. Louis, MO, USA). The analyses were performed using a Dionex UltiMate 3000 system (Dionex Corp., Sunnyvale, CA, USA) equipped with Thermo Q-Exactive (Thermo Fisher Scientific, Bremen, Germany). The separation was carried out on an Acclaim RSLC 120 C18 column (150 × 2.1 mm, 2.2 μm, Dionex Corp.). The mobile phase comprised acetonitrile (A) and 0.1% formic acid in water (B, v/v). Gradient elution was carried out as follows: 5% A, 0.0–1.0 min; 5.0–15.0% A, 1.0–5.0 min; 15.0–30.0% A, 5.0–12.0 min; 30–50% A, 12.0–15.0 min; 50–65% A, 15.0–17.0 min; 5–5% A, 17.0–22.0 min. The flow rate was 0.25 mL/min. The operation conditions in the MS/MS analysis were set as follows: ionization mode, positive; spray voltage, 4.0 kV; capillary temperature, 320°C; sheath gas pressure, 40 arbitrary units; auxiliary gas pressure, 10 arbitrary units; ion scans, 100–1,500 m/z; resolution of MS scans, 70,000; resolution of MS/MS scans, 17,500; and normalized collision energy, 10–70 eV. WEF (2.5–10 mg/mL) and mixtures (5–2,500 ng/mL) of authentic standards were prepared in methanol and filtered through a 0.22-μm filter membrane before injecting 3 μL aliquots for UPLC-MS/MS analysis. Scopoletin, as an internal standard, was used for quantitation of phytochemicals in WEF.

### Statistical analysis

One-way ANOVA with Dunnett's test was used for the comparison of two groups. Treatment effects were evaluated via analysis. Statistical analysis was performed using GraphPad PRISM software (GraphPad PRISM software Inc., Version 5.02, CA, USA). ^*^P < 0.01, ^**^P < 0.001, and ^***^P < 0.0001 indicated statistical significance.

## Results

### Effects of WEF on cytotoxicity in raw 264.7 cells

**Figure 1:**
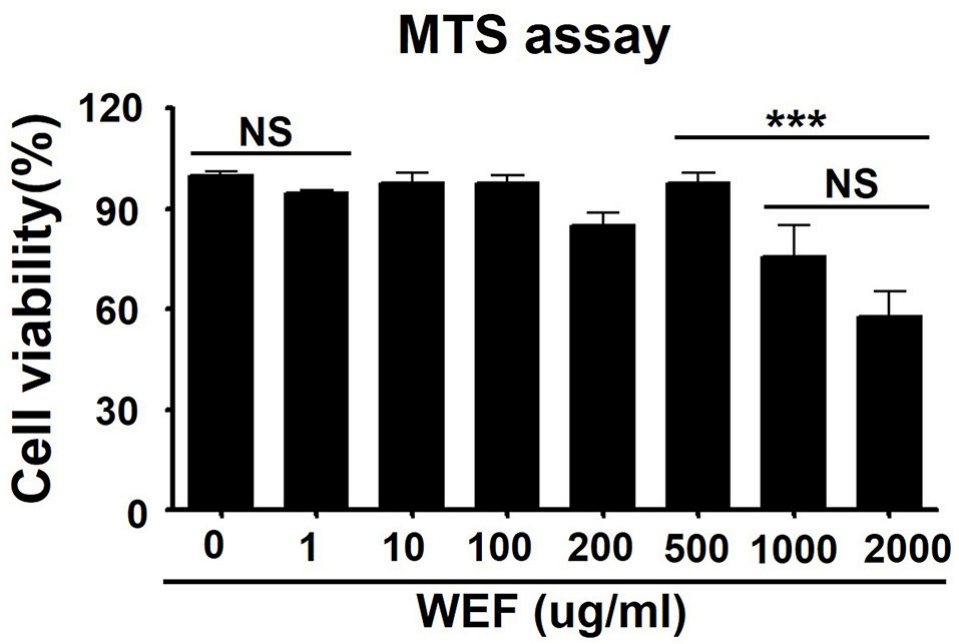
Determination of cytotoxicity and viability of water extract of *Eupatorium fortunei* (WEF) in RAW 264.7 cells. The viability of RAW 264.7 cells was determined by MTS assay after treatment with indicated concentrations of WEF for 24 h. Data are presented as the mean ± SD of three independent experiments. NS, no significance; ^***^P < 0.0001 vs. untreated cells.

The cytotoxicity of WEF was assessed using the MTS assay after 24 h treatment of murine RAW 264.7 macrophage cells with various concentrations of WEF. WEF at concentrations ≤500 μg/mL had no significant cytotoxicity on RAW 264.7 cells (Figure 1). Therefore, subsequent experiments investigating antiviral effects were performed using WEF at concentrations ≤100 μg/mL.

### Effects of WEF on pro-inflammatory cytokine production and type I IFN signaling pathway activation in RAW 264.7 murine macrophages

The pro-inflammatory cytokines and type I interferon play significant roles in inducing antiviral responses and immunoregulatory activities (Theofilopoulos et al., 2005). We performed a screening test to identify pro-inflammatory cytokines and observed the interferon-β production in traditional herbal medicines, and we found that WEF can be related to the innate immune response by inducing an antiviral response during the production of cytokines such as TNF-α, IL-6, and IFN-β.

**Figure 2:**
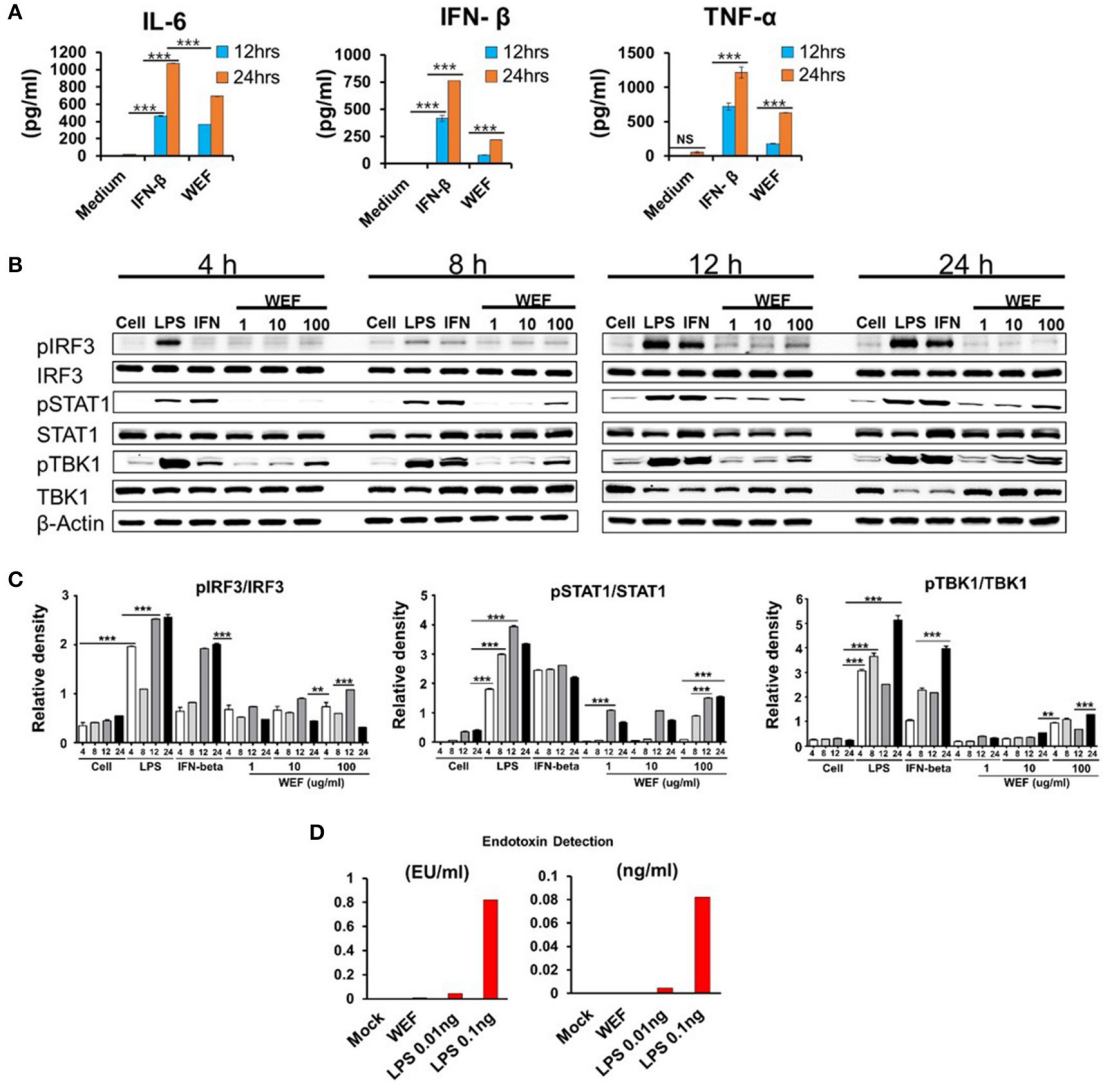
Induction of pro-inflammatory cytokines and activation of type I IFN by WEF *in vitro*. **(A)** RAW264.7 cells were treated with DMEM containing 10% FBS alone, 1,000 unit/mL recombinant mouse IFN-β, or 100 μg/mL WEF and incubated at 37°C with 5% CO_2_. Supernatant from each group was harvested at 12 and 24 h and clarified by centrifugation at 2,500 × g for 10 min at 4°C. Clarified supernatants were dispensed into murine IFN-β, TNF-α, and IL-6 capture antibody-coated ELISA plates in duplicate to measure cytokine secretion. **(B,C)** Western blot analysis was performed on whole cell lysates of macrophage-type cells treated with or without WEF (1, 10, and 100 μg/mL) to assess the expression of the non-phosphorylated and phosphorylated forms of IRF3, STAT1, TBK1, and β-actin over time. Similar results were obtained, and the experiment was performed three times independently. **(D)** Detection of contaminated endotoxin in WEF by Limulus Amebocyte Lysate (LAL) assay. (EU, endotoxin unit). ^***^P < 0.0001, ^**^P < 0.001 vs. untreated cells.

We investigated the effect of WEF on the secretion of TNF-α, IL-6, and IFN-β using ELISA. Concentrations of secreted IL-6, IFN-β, and TNF-α were 693.4167, 134.0662, and 629.625 pg/mL, respectively, after 24 h of WEF treatment (Figure 2A). These results indicate that IL-6, IFN-β, and TNF-α mediate the antiviral response induced by WEF in RAW 264.7 cells. The expression of phosphorylated and non-phosphorylated IRF3, STAT1, and TBK1 in WEF-treated RAW 264.7 cells were determined using Western blot analysis. The results indicate that WEF treatment upregulates the phosphorylation of IRF-3, STAT1, and TBK1, which are key molecules in the type I IFN signaling pathway (Figures 2B,C). Testing for contamination of WEF by endotoxin (LPS), a known immunomodulator, was negative according to the Limulus Amebocyte Lysate (LAL) assay (Figure 2D).

### WEF suppresses viral replication in raw 264.7 cells

**Figure 3:**
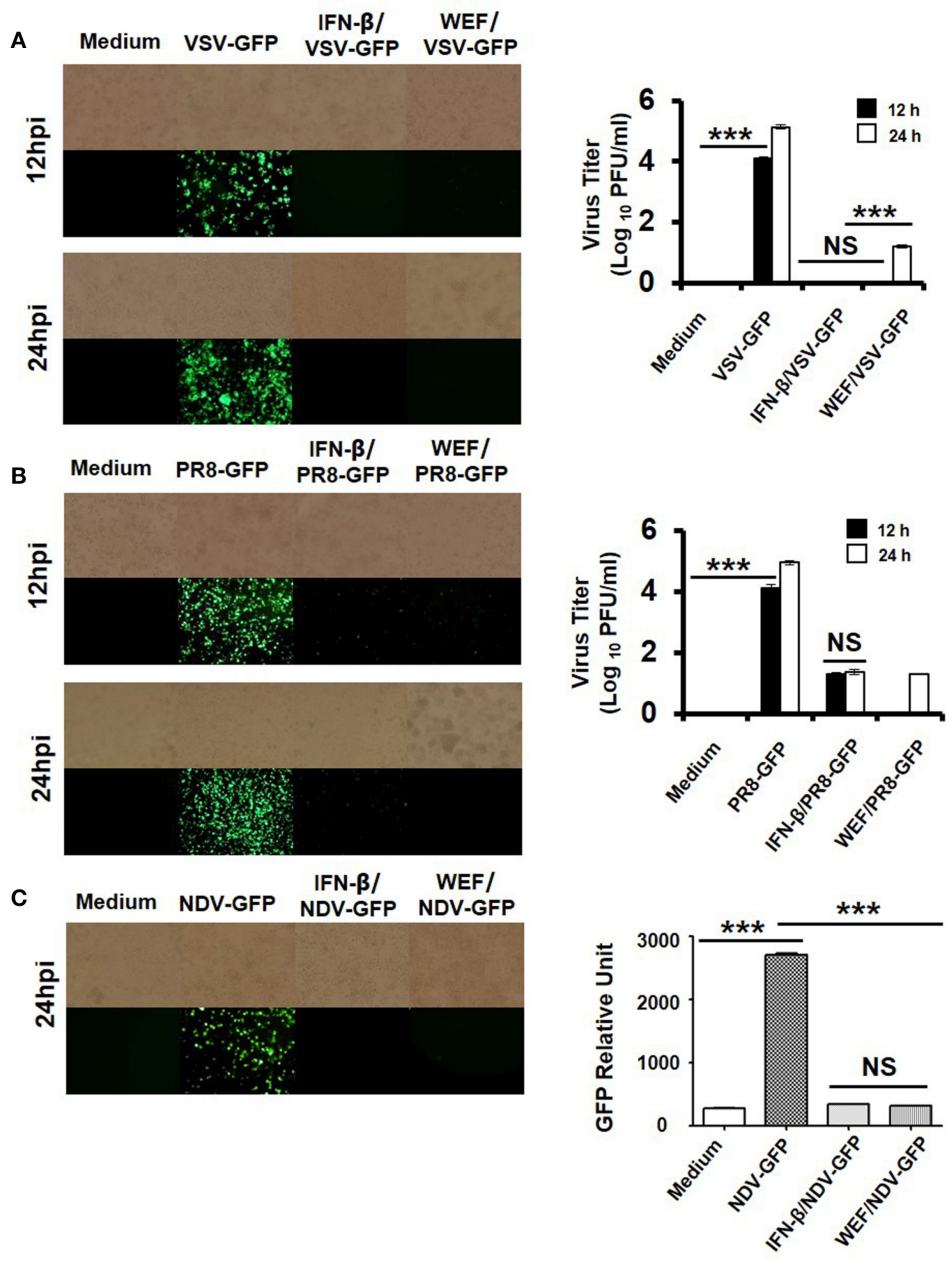
Antiviral activities of WEF in RAW 264.7 cells. Cells were treated with medium alone, 100 μg/mL WEF, or 1,000 U/mL recombinant mouse IFN-β 12 h prior to infection with **(A)** VSV-GFP, **(B)** PR8-GFP, or **(C)** NDV-GFP. Images were obtained at 24 hpi (200× magnification). Virus titers were determined from the supernatant for VSV-GFP and from the infected cells for PR8-GFP. NS, no significance; ^***^P < 0.0001 vs. untreated cells.

To investigate the antiviral activity of WEF, we assessed the suppression of viral replication using GFP-expressing VSV, PR8, and NDV viruses in RAW 264.7 cells. Viral replication was monitored via GFP expression. WEF-treated RAW264.7 cells exhibited significantly reduced GFP expression than untreated cells, which had high levels of GFP expression upon infection with VSV (Figure 3A), PR8 (Figure 3B), and NDV (Figure 3C). These results are consistent with the viral titers of GFP-expressing VSV, and PR8 viruses in the cell supernatant and infected cells. WEF treatment significantly reduced the viral titers by nearly 4.8-, and 4.2-folds against GFP-expressing VSV, and PR8, respectively, at 24 hpi (Figures 3A,B). Compared with untreated cells, WEF-treated cells displayed significantly less cell death following infection with all tested viruses.

### WEF treatment decreases influenza a virus M2 protein levels and inhibits H1n1 infection in RAW 264.7 cells

**Figure 4:**
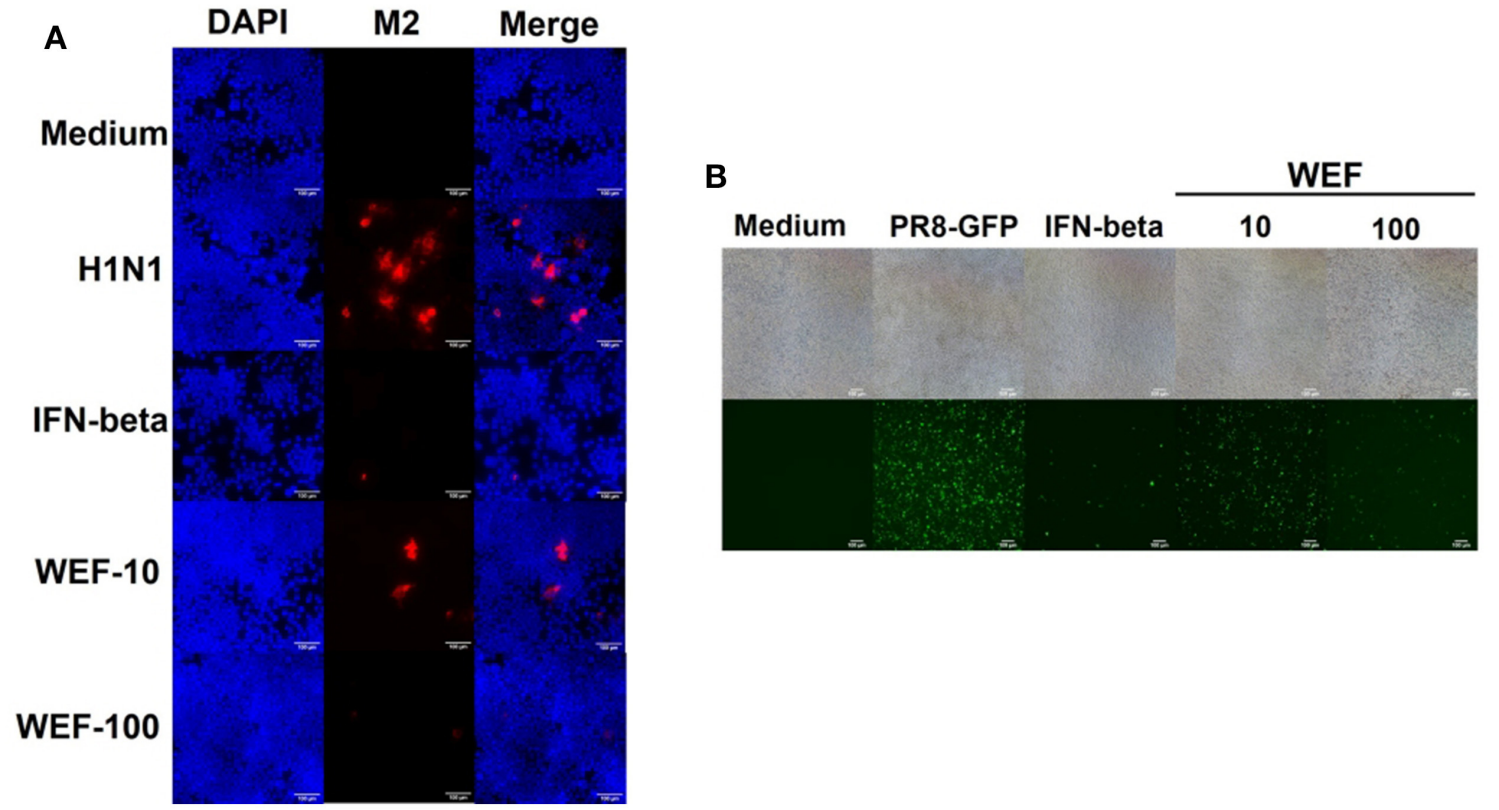
Effect of WEF on the growth influenza A/PR/8/34 (H1N1) virus in RAW 264.7 cells. **(A)** Immunofluorescence analysis of influenza A M2 protein in cells. Cells were treated with WEF (10, 100 μg/mL) and IFN-β (1,000 U/mL) after influenza A virus infection. Influenza A virus M2-specific antibody was used for detection by fluorescence microscopy. Cells were also stained with DAPI, and the merged image shows the cytoplasmic location of the virus M2 protein (red). **(B)** RAW 264.7 cells were treated with WEF (10, 100 μg/mL) and IFN-β (1,000 U/mL) 12 h before infection with PR8-GFP (MOI 1). The antiviral effect was verified after 24 h of viral infection as the presence or absence of GFP expression.

Immunofluorescence analysis demonstrated that WEF-treated cells expressed significantly less M2 protein than did untreated cells at 24 hpi (Figure 4A). The replication of GFP-expressing influenza (A/Puerto Rico/8/34) virus was significantly less in WEF-treated cells than in untreated RAW 264.7 cells (Figure 4B). Thus, these data indicate that WEF reduces influenza H1N1 viral protein expression and inhibition.

### Chemical composition of WEF by UPLC-MS/MS analysis

**Figure 5:**
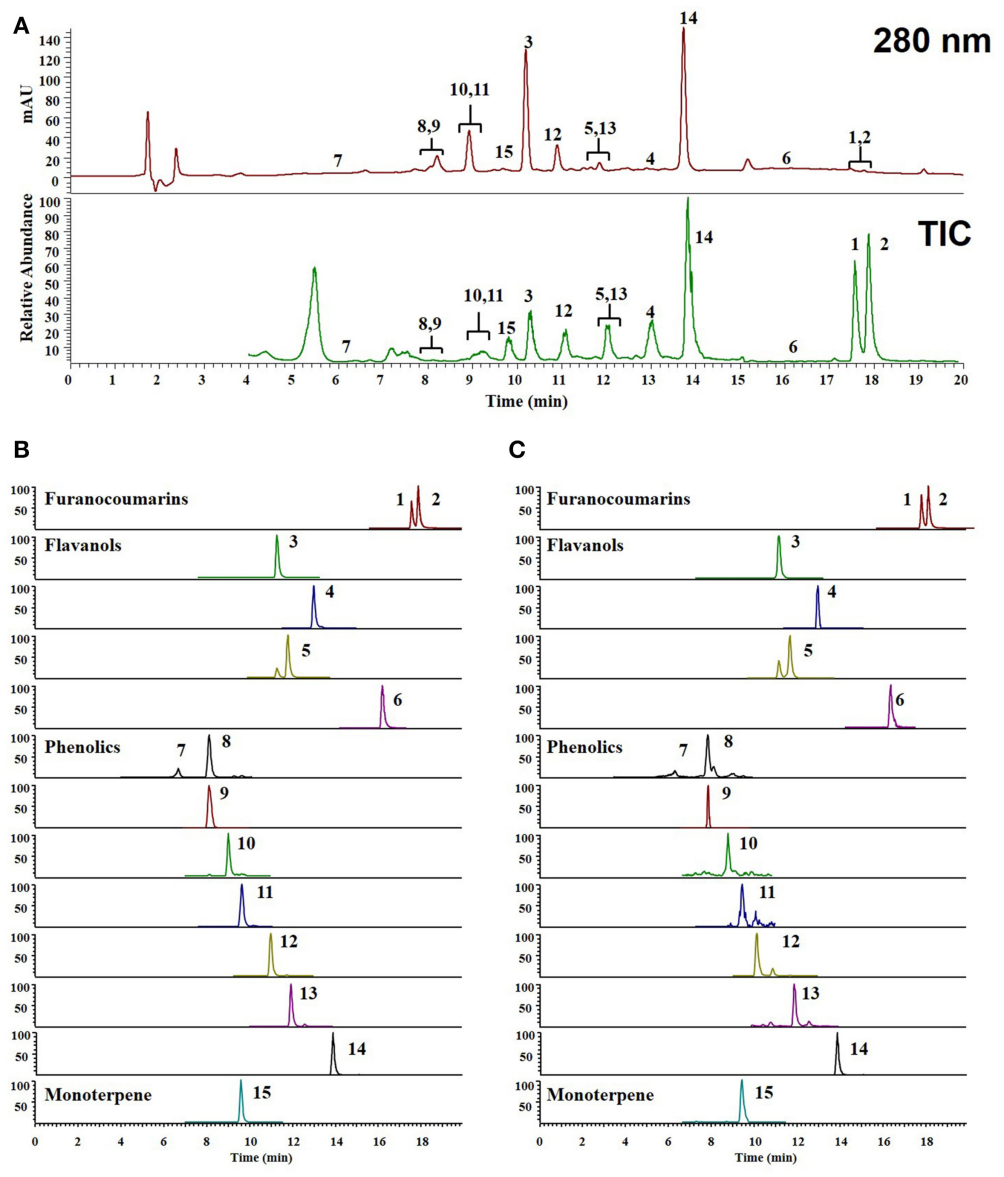
UPLC-MS/MS chromatograms of identified phytochemicals. **(A)** UV chromatogram and total ion chromatogram (TIC) of EF **(B)** parallel reaction monitoring (PRM) chromatogram of authentic standards, **(C)** PRM chromatogram of EF. psoralen (1), angelicin (2), rutin (3), quercitrin (4), isoquercitirin (5), quercetin (6), neochlorogenic acid (7), chlorogenic acid (8) syringin (9), caffeic acid (10), 1,3-dicaffeoylquinic acid (11), *p*-coumaric acid (12), ferulic acid (13), *o*-coumaric acid (14), and sweroside (15).

To identify the composition and effectiveness of the bioactive component, because of the chemical diversity and complexity of herbal and herbal derivatives, diversification of the extraction of raw materials and production methods are available. To identify the various components of WEF, this study analyzed various traditionally used water extracts. As a result of this study, UPLC-MS/MS analysis comparing retention time and mass fragmentation of authentic standards identified the following components of WEF: two coumarins (psoralen, and angelicin), four flavonoids (rutin, quercitrin, isoquercitrin, and quercetin), eight phenolics (neochlorogenic acid, chlorogenic acid, syringin, caffeic acid, 1,3-dicaffeoylquinic acid, *p*-coumaric acid, *o*-coumaric acid, and ferulic acid), and one monoterpene (sweroside) (Figure 5) (Table 2). The amounts of identified phytochemicals ranged from 0.002 to 0.557 mg/g extract. In this regard, the antiviral effect of WEF against RNA viruses may result from the beneficial effects of constituents identified in this study.

**Table 2:** Identification and quantitation of phytochemicals in water extract of *Eupatorium fortunei* (WEF) using UPLC- MS/MS analysis.

**No**	**t_R_ (min)**	**[M+H]^+^ (*m/z*)**		**Elemental composition**	**Error (ppm)**	**MS/MS fragments (m/z)**	Identification^*^	**Contents (mg/g)**
		**Calculated**						
**COUMARINS**								
1	17.58	187.03897	187.03905	C_11_H_6_O_3_	0.413	159.04413, 149.04915, 131.04925	Psoralen	0.022
2	17.89	187.03897	187.03906	C_11_H_6_O_3_	0.495	159.04413, 143.04914, 131.04923	Angelicin	0.011
**FLAVONOIDS**								
3	11.30	611.16066	611.11070	C_27_H_30_O_16_	0.674	465.10291, 303.04932, 147.06511, 129.05473	Rutin	0.017
4	11.02	449.10784	449.10779	C_21_H_20_O_11_	0.596	303.04977, 147.06514, 129.05476	Quercitrin	0.002
5	11.80	465.10275	465.10303	C_21_H_20_O_12_	−0.116	303.04980, 145.04956	Isoquercitrin	0.009
6	15.85	303.04993	303.04987	C_15_H_10_O_7_	−0.212	285.03949, 155.04890, 153.01811, 109.02841	Quercetin	0.008
**PHENOLICS**								
7	6.72	355.10236	355.10074	C_16_H_18_O_9_	−4.566	163.03891	Neochlorogenic acid	0.021
8	8.15	355.10236	355.10254	C_16_H_18_O_9_	0.504	163.03889	Chlorogenic acid	0.022
9	8.16	395.13125	395.13129	C_17_H_24_O_9_	0.093	233.07494, 185.04199, 85.02895	Syringin	0.030
10	9.05	181.04954	181.04962	C_9_H_8_O_4_	0.451	163.03885, 135.04406	Caffeic acid	0.209
11	9.71	517.13405	517.13348	C_25_H_24_O_12_	−1.095	499.12347, 337.09146, 319.08123, 163.03885	1,3-Dicaffeoylquinic acid	0.002
12	10.31	165.05462	165.05464	C_9_H_8_O_3_	0.132	147.04395, 119.04934	*p*-Coumaric acid	0.115
13	11.98	195.06519	195.06540	C_10_H_10_O_4_	1.072	177.05461, 163.03896,149.05971	Ferulic acid	0.036
14	13.85	165.05462	165.05470	C_9_H_8_O_3_	0.501	147.04406, 103.05469	*o*-Coumaric acid	0.557
**MONOTERPENE**								
15	9.65	359.13366	359.13367	C_16_H_22_O_9_	0.020	197.08078, 179.07008, 127.03909	Sweroside	0.013

* *compared with retention time and MS spectral data of authentic standards*.

### Antiviral activity of the 15 identified WEF components

**Figure 6:**
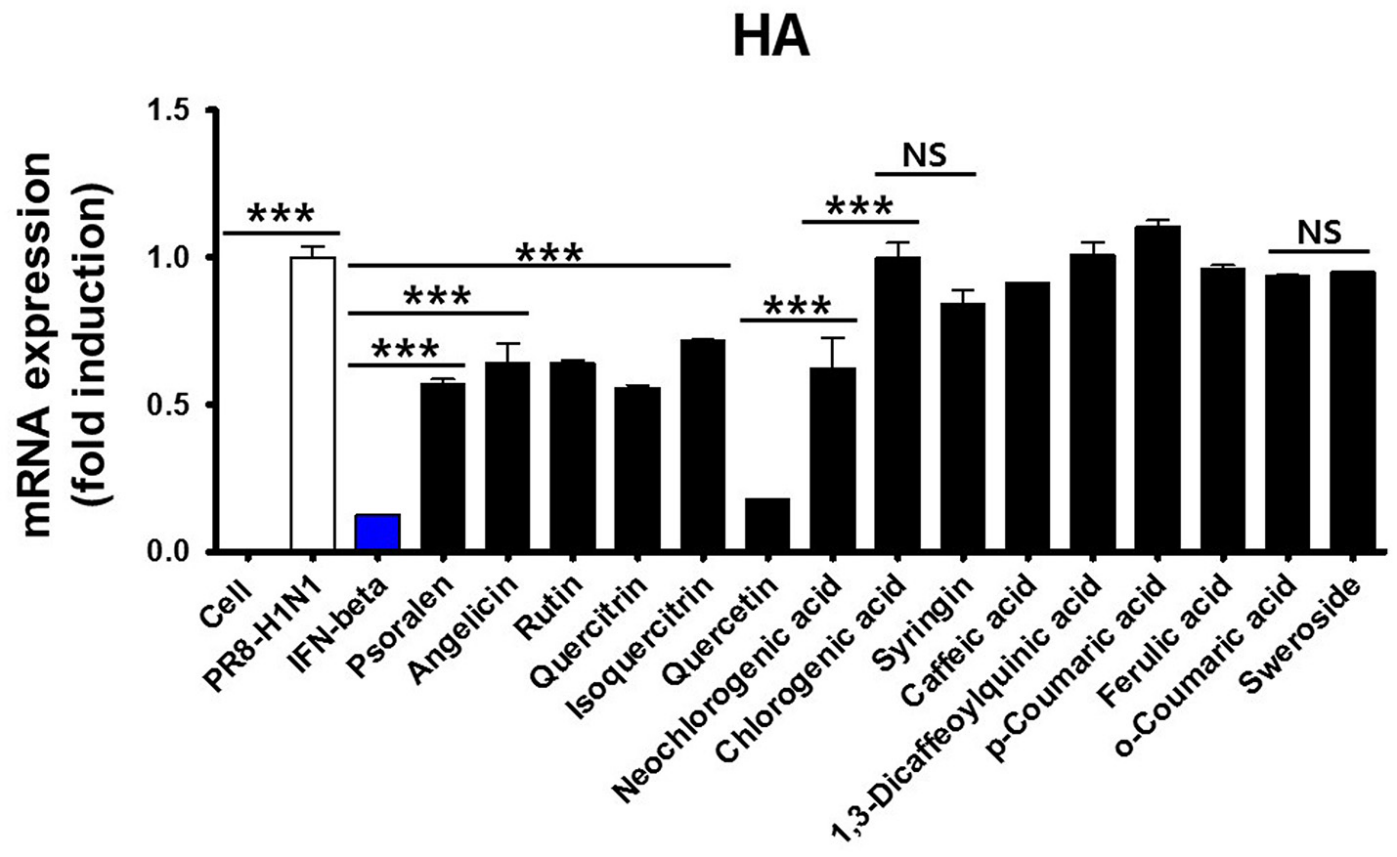
Effect of WEF constituents on influenza A/PR/8/34 HA mRNA synthesis. Pretreatment with 15 WEF components (0.2 μg/mL each) in influenza A/PR/8/34(MOI1)-infected RAW 264.7 cells and the relative levels of influenza A/PR/8/34 HA mRNA as analyzed by qRT-PCR and normalized to the levels of β-actin. NS, no significance; ^***^P < 0.0001 vs. untreated cells.

We first tested the antiviral activity of 15 components from WEF (Figure 6). The expression pattern of the influenza A virus gene HA was confirmed by qRT-PCR. Subsequently, 15 components were treated at a concentration of 0.2 μg/mL, A/PR/8/34 H1N1-infected cells were harvested, and the relative mRNA expression level of viral genes at 24 hpi was assessed by qRT-PCR. As a result, the expression of influenza A virus HA mRNA was lower than that of untreated cells after treatment with quercetin (0.81-fold), psoralen (0.314-fold), or quercitrin (0.334-fold) (Figure 6).

**Figure 7:**
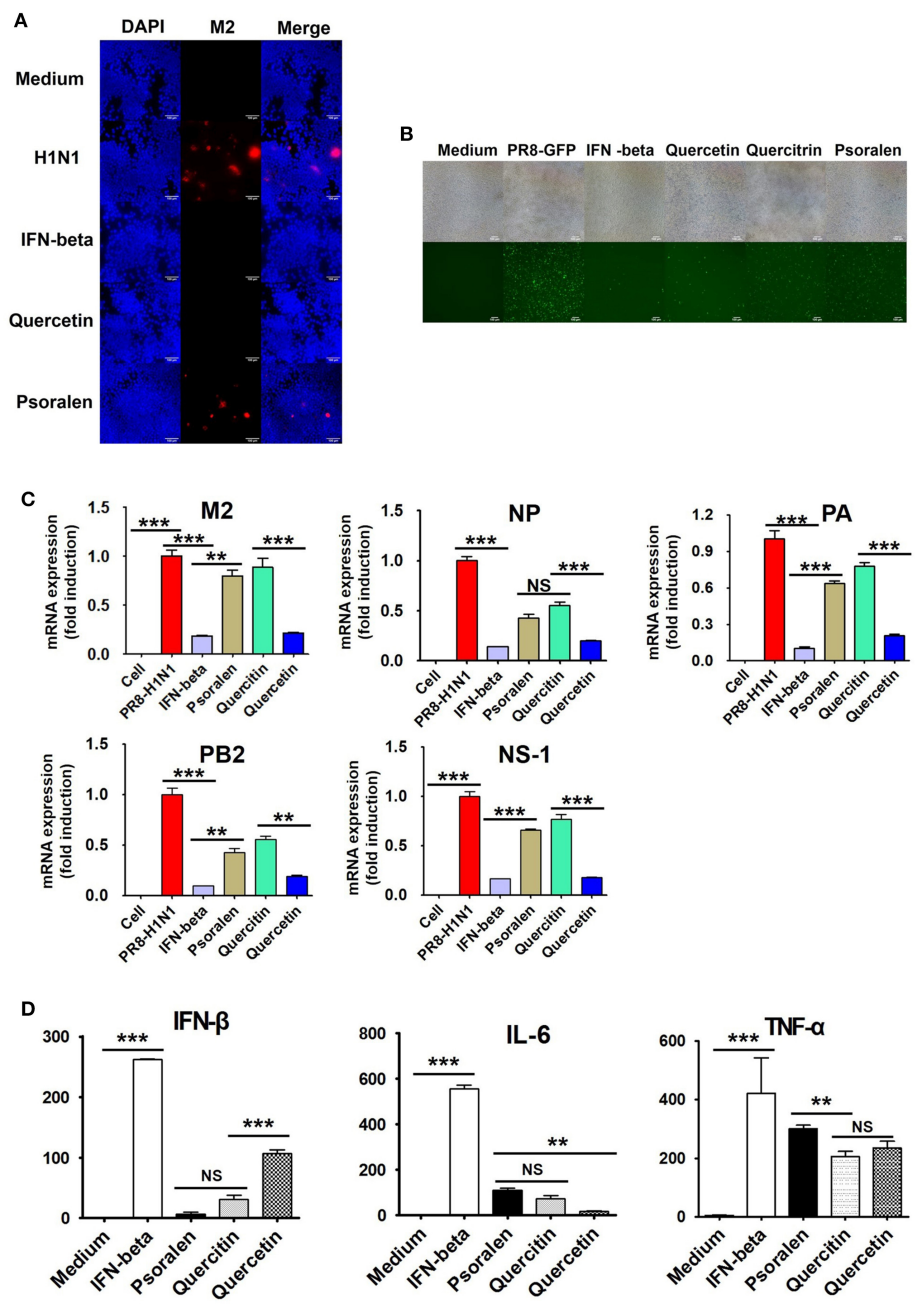
Effect of quercetin, psoralen, and quercitrin from WEF on the growth of influenza A/PR/8/34 (H1N1) virus in RAW 264.7 cells. **(A)** Cells were treated with quercetin, psoralen, or quercitrin (0.2 μg/mL) and IFN-β (1,000 U/mL) 12 h before infection with PR8-GFP (MOI 1). The antiviral effect was verified after 24 h of viral infection as the presence or absence of GFP expression. **(B)** Cells were treated with quercetin, psoralen, or quercitrin (0.2 μg/mL) and IFN-β (1,000 U/mL), after influenza A virus infection. Influenza A virus M2-specific antibody was used to observe M2 protein in cells by fluorescence microscopy. **(C)** Pretreatment of influenza A/PR/8/34(MOI1)-infected cells with 15 WEF components (0.2 μg/mL each) and the relative levels of influenza A/PR/8/34 M2, NP, PA, PB2 and NS-1 mRNA as analyzed by qRT-PCR and normalized to the levels of β-actin. **(D)** Induction of IFN-β cytokine *in vitro*. Cells were treated with DMEM containing 10% FBS alone, 1,000 U/mL recombinant mouse IFN-β, or 0.2 ug/mL of quercetin, psoralen, or quercitrin. Supernatants from each group were harvested at 24 h, clarified, and dispensed into murine IFN-β, TNF-α, and IL-6 capture antibody-coated ELISA plates to measure cytokine secretion. NS, no significance; ^***^P < 0.0001, ^**^P < 0.001 vs. untreated cells.

Next, we investigated the inhibition of influenza virus A/PR/8/34 replication as a function of H1N1 expression in RAW 264.7 cells using a GFP-expressing influenza virus. GFP expression was significantly reduced in cells treated with quercetin than in those treated with psoralen or quercitrin and in untreated cells (Figure 7A). Immunofluorescence analysis demonstrated that the expression of M2 protein in RAW 264.7 cells was suppressed in cells treated with quercetin than in those treated with psoralen or untreated cells (Figure 7B).

We further investigated whether quercetin, psoralen, and quercitrin treatment inhibits viral mRNA (M2, NP, PA, PB2, and NS-1) synthesis. A/PR/8/34 H1N1-infected cells were harvested and relative mRNA expression level of viral genes at 24 hpi were assessed by qRT-PCR. The synthesis of influenza mRNA for M2, NP, PA, PB2, and NS-1 was dramatically suppressed in quercetin-treated cells than in psoralen- and quercitrin-treated cells and in untreated cells (Figure 7C). We investigated the effect of quercetin, psoralen, and quercitrin on IFN-β secretion, as measured by ELISA. The concentration of secreted IFN-β, IL-6, and TNF-α after 24 h of treatment with quercetin was 107.21, 17.19, and 234.885 pg/mL, respectively (Figure 7D).

## Discussion

We observed here that the total WEF exerted a striking anti-viral effect on cells *in vitro*. We identified the active compounds present in WEF using UPLC-MS/MS and confirmed that quercetin, psoralen, and quercitrin exhibited antiviral effects. Based on the above experimental results, the antiviral effect and immune-regulatory properties of quercetin were investigated. Quercetin, psoralen, and quercitrin showed significantly inhibited viral replication and induced cytokine secretion, such as IFN-beta. Thus, the observed antiviral activity of WEF may be exerted, at least in part, by the quercetin, psoralen, and quercitrin in the WEF.

The role of immunity, which is the ability of the human body to defend against infectious diseases, is becoming more important (Frieman et al., 2008) as new infectious diseases, such as avian influenza virus, Middle East Respiratory Syndrome (MERS), and Acute Respiratory Syndrome (SARS) infection, have emerged (Morens and Fauci, 2013). Because the occurrence of viral infection mainly occurs in a state of deteriorated immune function, various studies have been conducted to find natural products capable of enhancing these immune responses (Matsumoto et al., 2010; Wang et al., 2012; Singh et al., 2015; Choi et al., 2016).

Macrophages play a role in rapidly transmitting defense mechanisms against viral infections. When macrophages are activated during inflammatory reactions, they produce many inflammatory factors, including tumor necrosis factor-α (TNF-α), interleukin-6 (IL-6), and interferon-β (IFN-β) (Ellermann-Eriksen, 2005; Theofilopoulos et al., 2005; Melchjorsen et al., 2006). These pro-inflammatory cytokines and type I interferon play an important role in inducing antiviral responses and immunoregulatory activities (Konopka et al., 2009; Paludan et al., 2011). IFN-β continuously induces the synthesis of antiviral proteins, inhibiting intracellular viral replication and promoting adaptive immunity against viruses (Yoneyama et al., 2005). In this study, we sought to elucidate the mechanism underlying WEF antiviral activity. We observed that WEF treatment induces high levels of IL-6, IFN-β, and TNF-α secretion (Figure 2A) compared with untreated cells after 24 h. WEF-induced IL-6, IFN-β, and TNF-α may then mediate the antiviral state in RAW 264.7 cells. We also observed that WEF treatment induces increased phosphorylation of IRF3, STAT1, and TBK1 relative to untreated cells after 12 h (Figure 2B).

We observed that WEF protects RAW 264.7 cells against infection by the RNA viruses VSV, NDV, and influenza A. Antiviral activity was monitored as decreases in GFP expression in cells exposed to GFP-labeled viruses. WEF-treated RAW264.7 cells exhibited markedly lower GFP expression than untreated cells, which expressed high levels of GFP for the RNA viruses VSV, PR8, and NDV (Figures 3A–C). These results are consistent with the titers of VSV, NDV, and PR8 in the supernatants of the infected cells. WEF treatment decreased the titers of VSV and PR8 by nearly 4.2-fold, and 3.8-fold, respectively (Figures 3A,B). These results clearly indicate that the WEF can inhibit the replication of these RNA viruses in RAW 264.7 cells.

The underlying mechanism of WEF antiviral activity likely involves the induction of type I IFN and pro-inflammatory cytokine secretion by WEF components, including quercetin, psoralen, and quercitrin, leading to an antiviral state in the host cell. WEF is thus a promising prophylactic agent for inhibiting viral infections through the activation of innate immunity. Several limitations of this study should be acknowledged. The optimal dosage of WEF for practical application and the duration of the effects within the host should be examined to obtain the best possible protection against viral infections. In addition, this study investigated the anti-viral activity of WEF and its components only *in vitro*. Therefore, further extensive studies are needed to assess the potential of WEF and its components for use as *in vivo* antiviral therapies. In addition, studies to determine the specific signaling pathways involved in WEF action and the role of interactions between WEF components in its antiviral activity are also needed.

The genus *Eupatorium* (family Asteraceae) is known to include ~1,200 species in the world including Asia, America, Africa, and Europe (Liu et al., 2015) and are known to have many biological activities including anti-nociceptive, anti-inflammatory, antibacterial, antifungal, anticancer, antiplasmodial, antioxidative, and anti-allergic activities and immunomodulating properties (Liu et al., 2015). The chemical composition of *Eupatorium* species include bioactive compounds such as flavonoids, monoterpene derivatives, sesquiterpenes, diterpenes, triterpenes, pyrrolizidine alkaloids, essential oil, and some other components (Liu et al., 2015).

In addition, extracts of some *Eupatorium* species and their components have been reported to exhibit antiviral effects; for example, euparin isolated from *E. chinense* exhibits antiviral activities against respiratory syncytial virus (Wang et al., 2016), the hydroalcoholic extract of *E. perfoliatum* exhibits antiviral activities against influenza A virus that are mediated by interaction with the viral hemagglutinin (Derksen et al., 2016), *E. adenophorum* polysaccharide confers protective effects against H5N1 influenza infection (Jin et al., 2013), euparin from *E. buniifolium* exhibits antiviral activities against poliovirus type 1 (Visintini Jaime et al., 2013) and pseudorabies virus strain RC/79 (Zanon et al., 1999), and essential oils of *E. patens* inactivates HSV-1 (Garcia et al., 2003). Furthermore, extracts of *E. buniifolium* and *E. articulatum* exhibited antiviral effects against HSV-1 and VSV, respectively, and *E. glutinosum* inhibits VSV replication (Abad et al., 1999).

In this study, WEF component analysis and activity test showed that quercetin had an excellent antiviral effect against influenza A virus. It has been reported that quercetin components are present in *E. odoratum* (Yuan et al., 2007), *E. lindleyanum* (Qian et al., 2004), and *E. ballotaefolium* (Militao et al., 2004), and these plants may have antiviral efficacy. Also, according to recent research results, quercetin has been shown to protect against the entry of influenza A virus (Wu et al., 2015) and interaction between human respiratory syncytial virus M2-1 protein (Teixeira et al., 2017), have anti-HSV-1 and anti-HSV-2 properties (Lee et al., 2017), and inhibit coronavirus and dengue virus infection (Chiow et al., 2016). Thus, *E. odoratum, E. lindleyanum*, and *E. ballotaefolium* have possible antiviral effect on various RNA viruses.

In conclusion, this study is the first to demonstrate the efficacy of WEF as an alternative antiviral agent. WEF inhibited VSV, NDV, and PR8 replication in murine macrophages via induction of pro-inflammatory cytokines and Type I IFN signaling, leading to an antiviral state. WEF or its components, including quercetin, psoralen, and quercitrin, can be useful as therapeutic or preventive agents to limit viral replication. Furthermore, WEF and its components may be useful starting materials for the development of potent herbal agents against selected viral infections.

## Author contributions

Designed study and revised manuscript: JM, WC, and JC. Performed experiments: JL, HL, YH, and JC. Analyzed data: JC, YH, and HL. Wrote manuscript: JC.

### Conflict of interest statement

The authors declare that the research was conducted in the absence of any commercial or financial relationships that could be construed as a potential conflict of interest.
